# Presynaptic localization of GluK5 in rod photoreceptors suggests a novel function of high affinity glutamate receptors in the mammalian retina

**DOI:** 10.1371/journal.pone.0172967

**Published:** 2017-02-24

**Authors:** Iris Haumann, Dirk Junghans, Max Anstötz, Michael Frotscher

**Affiliations:** 1 Institute of Neuroanatomy, University Medical Center Hamburg-Eppendorf, Hamburg, Germany; 2 Institute of Embryology and Stem Cell Biology, Department of Biomedicine, University of Basel, Basel, Switzerland; 3 Institute for Structural Neurobiology, Center for Molecular Neurobiology Hamburg (ZMNH), University Medical Center Hamburg-Eppendorf, Hamburg, Germany; Institut de la vision, FRANCE

## Abstract

Kainate receptors mediate glutamatergic signaling through both pre- and presynaptic receptors. Here, we studied the expression of the high affinity kainate receptor GluK5 in the mouse retina. Double-immunofluoresence labeling and electron microscopic analysis revealed a presynaptic localization of GluK5 in the outer plexiform layer. Unexpectedly, we found GluK5 almost exclusively localized to the presynaptic ribbon of photoreceptor terminals. Moreover, in GluK5-deficient mutant mice the structural integrity of synaptic ribbons was severely altered pointing to a novel function of GluK5 in organizing synaptic ribbons in the presynaptic terminals of rod photoreceptors.

## Introduction

L-Glutamate is the major excitatory neurotransmitter in the brain. Glutamatergic signals are mediated by metabotropic and ionotropic glutamate receptors. Based on their pharmacological properties the ion channel forming glutamate receptor family has been sub-divided into Kainate-, NMDA- and AMPA-type of ionotropic glutamate receptors [1, 2] with NMDA- and AMPA-type receptors being best known to mediate postsynaptic currents at excitatory synapses throughout the nervous system [3]. In contrast, the physiological properties of kainate receptors [4, 5] and their precise role in synaptic transmission are much less understood [6–8]. The discovery of selective antagonists, which enabled the isolation of kainate-receptor mediated currents [9–11], as well as their cloning and molecular characterization identified kainate receptor subunits as being distinct from subunits that contribute to AMPA and NMDA receptors [1, 12–17]. Five different kainate receptors subunits have been identified so far [1]. Based on sequence homologies and agonist binding properties they can be subdivided into the low-affinity kainate receptor subunits GLUK1, GLUK2 and GLUK3 [18–21] and the high-affinity subunits GLUK4 and GLUK5 [22–24]. Both families of kainate receptor subunits display weak homology with subunits of NMDA (10–20%) and AMPA receptors (30–35%).

Virtually all presynaptic terminals in the central nervous system express neurotransmitter receptors and it is thought that these presynaptic receptors are mainly of the metabotropic type [25, 26]. Metabotropic glutamate receptors are G-protein coupled receptors that upon ligand binding activate intracellular second messenger systems [27]. Growing evidence suggests that presynaptic ionotropic receptors are more widespread than previously believed [28] and contribute to presynaptic function such as neurotransmitter release [29–33]. Although the exact localization of presynaptic kainate receptors (KARs) and the mechanism by which they modulate neurotransmitter release have remained controversial, evidence for the existence of presynaptic kainate receptors has been available for some time [34–36]. Overlapping expression patterns of individual kainate receptor subunits indicate diverse subunit composition of native kainate receptors. Nevertheless, it is not clear whether kainate receptor subunit composition relates to their subcellular localization in neurons.

A key feature of the mammalian retina is its processing of the visual signal in parallel channels. Two of these parallel pathways are the so-called ON- and OFF-channels, which are transmitting light and dark signals. An anatomical basis for this parallel processing can be seen in the different types of retinal neurons and their connections. The mammalian retina is organized in layers—three cell layers and two synaptic layers. Rod and cone photoreceptors are located in the outer nuclear layer (ONL), whereas horizontal cells, bipolar cells, amacrine cells and Müller cells are located within the inner nuclear layer (INL). Retinal ganglion cells and a subset of amacrine cells contribute to the ganglion cell layer (GCL). In the mammalian retina glutamate mediates the synaptic transfer from photoreceptors to bipolar and horizontal cells in the outer plexiform layer (OPL) and from bipolar to amacrine cells and ganglion cells in the inner plexiform layer (IPL) [37, 38]. Retinal glutamate acts on both, ionotropic and metabotropic glutamate receptors. In the retina, metabotropic glutamate receptors predominate in the ON-channel pathway, whereas ionotropic glutamate receptors are used in the OFF-channel. As for the expression of KARs in the retina, much is known from RNA *in situ* hybridization studies [39–41]. Because of the limited antibody tools available to study kainate receptor expression and function, a detailed description of their localization in the mammalian retina is still missing. Accordingly, there is still uncertainty regarding the subunit distribution of kainate receptors within the mammalian retina at the sub-cellular level.

Here, we studied the expression of the kainate receptor subunit GluK5 (synonym: KA2) in the outer part of the mouse retina at light- and electron microscopic levels by using a GluK5 specific antiserum. In addition, we examined the retinal ultrastructure of wild-type (wt) and GluK5-deficient mutant mice. Our analysis revealed an unexpected presynaptic localization of GluK5 in rod photoreceptors terminals. In contrast to other known glutamate receptor subunits we found GluK5 as being localized almost exclusively in close proximity to the synaptic ribbon. Ultrastructural analysis of GluK5-deficient animals revealed alterations of synaptic ribbon architecture suggesting a novel function of GluK5.

## Materials and methods

### Animals and tissue preparation

Experiments were performed in agreement with the German law on the use of laboratory animals and the institutional guidelines of the University of Freiburg and the animal welfare office of the University of Freiburg. Retinae were prepared from adult wild type (WT) and GluK5^-/-^ animals [42]. WT control animals were of C57b/6 or hybrid 129SvEv/C57b/6 background. GluK5^-/-^ mutant animals were kept on 129SvEv/C57b/6 hybrid background. The animals were kept in an artificial day/night rhythm and were sacrificed in the morning of the artificial day. Mice were anesthetized with CO_2_ and killed by cervical dislocation. 5 WT (hybrid background), 3 WT (C57b/6) and 5 mutant animals were used in of which one eye was utilized for light microscopy and the other for electron microscopy. For dark adaptation 2 WT mice were dark-adapted for 3–4 hours prior to the experiment. The animals were killed with CO_2_ and then the eyes were removed under infrared illumination.

For light microscopy using cryostate sections, the preparation of retinal tissues was performed as described previously [43]. Briefly, the eyes were enucleated and opened along the ora serrata. The anterior segment and vitreous body were removed, and the posterior eyecups with the retinae attached were immersion-fixed for 15–30 min in 4% paraformaldehyde (PFA) in phosphate buffered saline (PBS, pH = 7.4). Following fixation, the retinae were dissected from the eyecup and cryoprotected in graded sucrose (10%, 20% and 30% in PBS). Pieces of retinae were mounted in freezing medium (TissueTEK), sectioned vertically (12 μm) using a cryostat (Leica), and finally collected on slides.

For paraffin sectioning, 4–5 small holes were introduced into the anterior eyecup using a fine needle and the whole eye immersion-fixed with 4% PFA (in PBS, 4°C) for 2–4 days. The tissue was subsequently dehydrated in an increasing series of ethanol, incubated in xylol, embedded in paraffin, and 7 μm microtome sections (Leica) collected on slides [44].

For electron microscopy (EM) the posterior eyecups were fixed with 4% PFA and 2.5% glutaraldehyde in PBS for 60 min. Sectioning and processing was performed as described previously [45]. Briefly, after incubation in osmium tetroxide (1% in 0.1 M phosphate buffer, PB) for 40 min at room temperature in the dark, sections were washed in PB and double-distilled water and contrasted in 1% uranyl-acetate for 40 min. Sections were dehydrated in a series of ethanol, followed by propylene oxide. After 1h incubation in a propylene oxide-epoxy resin mixture (Durcopan ACM; Sigma-Aldrich; 1:1), sections were incubated in Durcopan overnight and flat embedded. After polymerization at 60°C, sections were cut at 50 nm thickness using an ultramicrotome (Reichert Ultracut E; Leica). Electron micrographs were taken on a LEO 906 electron microscope (Zeiss, Germany).

For EM pre-embedding immunolabelling the posterior eyecups were fixed in 4% PFA in PB (0.1 M, pH = 7,4). Because of the sensitivity of the antibody to fixation, no glutaraldehyde was used. The retinal tissue was fixed for 10 min only to preserve antigenicity. Small pieces of retina were embedded in agar, vertical vibratome sections (70 μm, Leica) were cut and subsequently subjected to pre-embedding EM immunohistochemistry.

### Immunohistochemistry

Fluorescence immunohistochemistry was performed as previously described (Hack et al., 2001; Junghans et al., 2008). For cryostat sections non-specific binding sites were blocked for 1 h at RT (10% normal goat serum (NGS), 0.5% Triton X-100 in PBS). The tissue was subsequently incubated overnight at 4°C with the primary antibodies (3% NGS, 0.5% Triton X-100 in PBS). After several washes in PBS sections were incubated for 1 h with fluorochrome-coupled secondary antibodies (3% NGS in PBS), washed in PBS and mounted (Kaisers glycerol gelatin, Merck).

Immunohistochemistry on paraffin sections including antigen retrieval was performed as previously described (Junghans, 2008). Peanut agglutinin (PNA) labeling of cone pedicles was performed as described previously [46].

Fluorescence imaging was done using an Axioscop2 and Axiovert 200M with ApoTome function (Zeiss, Germany) and images processed by using Zeiss AxioVision software.

### Pre-embedding immuno-electron microscopy

Ultrastructural localization of GluK5 using the GluK5 antibody was achieved by pre-embedding immuno-electron microscopy as previously described in detail [47]. Briefly, after blocking (10% NGS in PB), vibratome sections were incubated with the KA2 antiserum for 4 days at 4°C (1:500, 3% NGS in PBS). After several washes in PB sections were incubated with biotinylated secondary antibodies (1:100) for 2 hours. Next, sections were processed using the ABC method according to the manufacture’s protocol (Vectastain Elite ABC kit, Vector Laboratories, Inc). After washing with PB and 0.05 M Tris-HCl (pH = 7.6) visualization was achieved by incubating the sections in 3,3′-diaminobenzidine (DAB; 0.05% in 0.05 M Tris-HCl, pH = 7.6; 10 minutes), and finally incubating in DAB in the presence of H_2_O_2_ (0.05% DAB, 0.01% H_2_O_2_ in 0.05 M Tris-HCl, pH = 7.6). The reaction was stopped by rinsing the sections with Tris-HCl buffer solution (0.05 M Tris-HCl, pH = 7.6). Afterwards, sections were incubated in cacodylate buffer (0.1 M, pH = 7.4), post-fixed with 2.5% glutaraldehyde in cacodylate buffer for 2 hours at 4°C, and placed in cacodylate buffer overnight. After washing of the sections with distilled water, the DAB reaction product was silver-intensified by incubating the sections in a solution containing 2.6% hexamethylenetetramine, 0.2% AgNO_3_, and 0.2% sodium borate (10 minutes at 60°C). The sections were post-fixed in 0.05% OsO4 in cacodylate buffer and, after dehydration, flat-embedded in glycidether-100 based resin (Serva, Heidelberg, Germany). Ultrathin sections were cut and stained with uranyl acetate and lead citrate. The grids were finally examined and photographed on a Zeiss EM10 electron microscope.

### Antibodies

The following antibodies were used: rabbit anti-KA2 [42, 48] (generous gift by S. Heinemann and M.Darstein); mouse anti-synaptophysin (Synaptic Systems, Germany), mouse anti-CtBP2 (Becton Dickinson, Germany), mouse anti-Calbindin (Swant, Switzerland), mouse anti-protein-kinase C (Sigma, Germany), Fluorescein labeled peanut agglutinin (Vectorlabs Burlingame, CA, 200μg/ml), Alexa 647 coupled anti-CACNA1F, (Bioss, USA), Alexa-coupled secondary antibodies (Molecular Probes, Invitrogen),.

## Results

### GluK5 is strongly expressed in the IPL and OPL of the mouse retina

**Fig 1:**
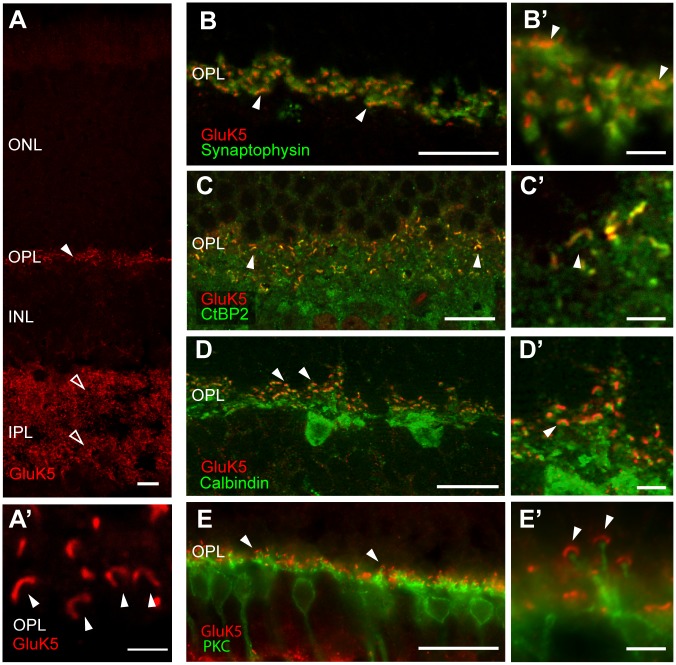
Immunohistochemical analysis of the mouse retina using an anti-GluK5 antibody. (A) Immunofluorescence analysis showing strong, punctate anti-GluK5 staining in the outer (arrowheads) and inner (open arrowheads) plexiform layer of the wild-type mouse retina. In the OPL (A’) the horseshoe-like appearance of the photoreceptor ribbons is clearly visible (arrowheads). (B) GluK5 immunolabeling does not colocalize with the presynaptic marker synaptophysin in the outer plexiform layer. High power photographs (B’) demonstrate adjacent localization (arrowheads). (C) Fluorescence immunostaining for CtBP2 and GluK5 in the outer plexiform layer. The horseshoe-like appearance of the photoreceptor synaptic ribbons colabels for CtBP2 and GluK5 and is clearly visible at higher magnification (arrowheads in C and C’). D) Double fluorescence immunolabeling for anti-GluK5 and anti-Calbindin, a marker for horizontal cells. The high-power photograph in D’ shows only very weak colocalization (arrowhead). (E) Double fluorescence immunolabeling with anti-PKC labeling rod bipolar cells and GluK5. GluK5 expression in the OPL does not colocalize with processes and terminals of rod bipolar cells (see higher magnification in E’). Scale bars: A—E: 10 μm; A’–E’: 2 μm.

In the present study we took advantage of a rabbit polyclonal antiserum raised against a synthetic C-terminal peptide of GluK5 that has previously been used to analyze the distribution of GluK5 in the mouse brain [34]. Fluorescence immunostaining revealed a strong labeling of GluK5 in the inner and outer plexiform layer of the retina (Fig 1A). In both plexiform layers a punctate staining reminiscent of a synaptic localization of GluK5 was seen (Fig 1A and 1A’). No or only very weak immunofluorescence was observed in the nuclear layers or ganglion cell layer. In order to verify the specificity of the anti-GluK5 immune-serum we performed immunostaining on retina sections of GluK5 deficient animals and did not observe any specific staining (S1 Fig).

### Presynaptic localization of GluK5 in the OPL

By performing immunofluorescence labeling using anti-GluK5 we observed numerous horseshoe-like structures in the OPL indicating a presynaptic localization of GluK5, possibly in association with synaptic ribbons (Fig 1A and 1A’). To test for a presynaptic localization of GluK5 in photoreceptor terminals we performed double labeling experiments with anti-GluK5 and anti-synaptophysin (Fig 1B and 1B’) and observed only a partial overlap of GluK5 and synaptophysin. The labeling for GluK5 appeared very localized in comparison to the diffuse staining for anti-synaptophysin. Since the horseshoe-like GluK5 labeling was mainly located presynaptically in the OPL, we performed double labeling with anti-CtBP2, a RIBEYE homologue known to label the synaptic ribbon structure [49, 50]. We observed most of the Gluk5 labeling overlapping with or in very close proximity to CtBP2 (Fig 1C and 1C’), indicating an association of GluK5 with the presynaptic ribbon structure in the OPL.

Next, we analyzed whether GluK5 is localized to horizontal cells. High affinity kainate receptors such as GluK5 are known to form heteromeric channels with low affinity subunits, some of which being localized presynaptically in processes of horizontal cells. Therefore, we performed double immunelabeling of horizontal cells using anti-GluK5 and anti-calbindin and observed GluK5 immunoreactivity adjacent to calbindin-positive structures with only very little colocalization (Fig 1D and 1D’). Similarly, we observed only rarely labeling for GluK5 in processes of horizontal cells when performing pre-embedding immunostaining for electron microscopy (EM). In view of the observed labeling in the OPL, Gluk5 could also be located to neuronal processes associated with the terminals of cone photoreceptors (inner part of the OPL, Fig 1B). Therefore, we performed PNA-labeling to identify cone terminals and their contacts with dendrites of OFF cone bipolar cells [51]. We found GluK5 immunoreactivity in close association with PNA labeled cone pedicles (S1B and S1B’ Fig). We did not observe double labeling using antibodies against GluK5 and glutamine synthetase, known to label Müller glia cells or the rod-bipolar cell marker protein kinase C (Fig 1E and 1E’). The major localization of GluK5 in the outer plexiform layer of the mouse retina is likely to be presynaptic and, importantly, closely associated with the synaptic ribbon. To test whether the pattern of expression is affected by light / dark adaptation, we dark-adapted WT animals for several hours before removing the eyes under infrared illumination. However we could not see a difference of GluK5 expression in these dark-adapted retinae.

It has been shown that the sites of calcium influx are adjacent to the synaptic ribbon [52] and that Ca^2+^ channels cluster near the ribbons [53, 54]. Accordingly, we performed co-labeling studies with anti-GluK5 and anti-CACNA1F to detect the calcium voltage-gated channel subunit alpha1 F and found both proteins localized adjacent to each other. A complete colocalization was not observed (S1C and S1C’ Fig).

### EM analysis confirms association of GluK5 with the synaptic ribbon

**Fig 2:**
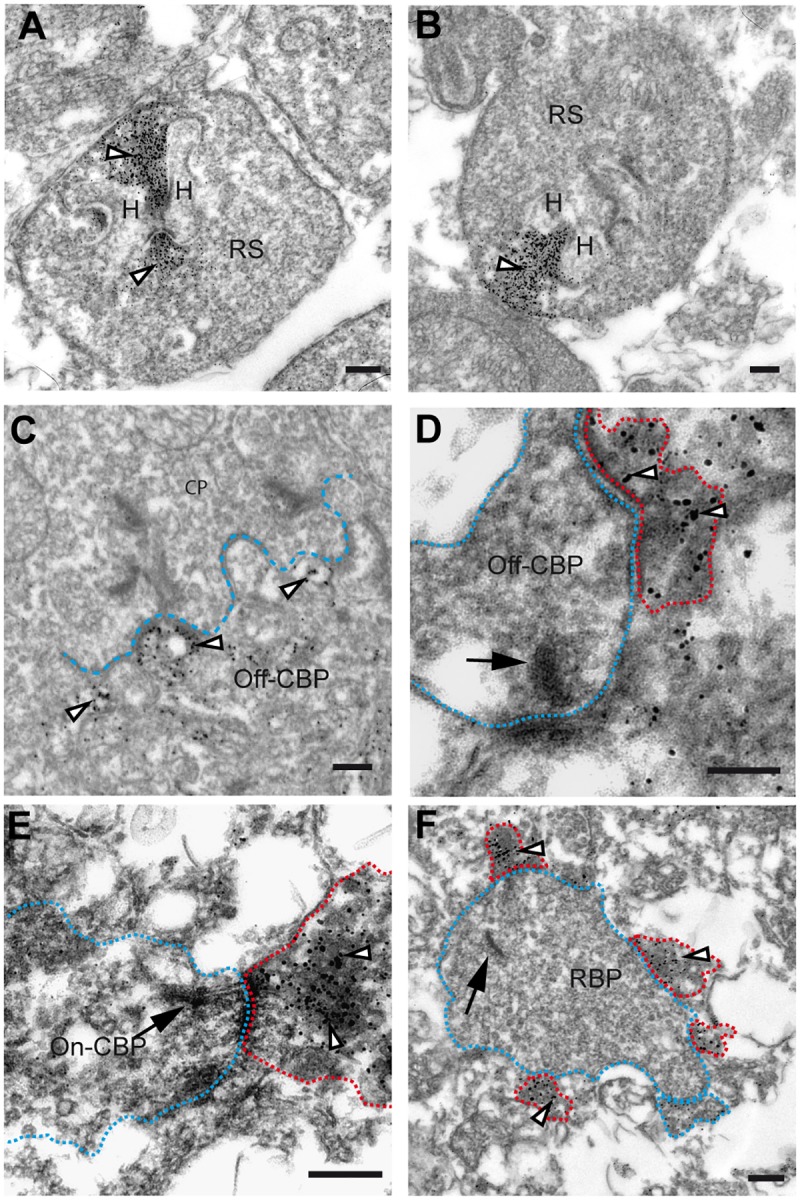
Ultrastructural localization of GluK5 in the outer and inner plexiform layer of the mouse retina. (A and B) Electron micrograph of rod photoreceptor ribbons showing the ultrastructural localization of GluK5 immunoreactivity in rod spherules. Note the distribution of GluK5 immunoreactivity at the rod photoreceptor ribbon synapse covering the entire presynaptic ribbon structure (arrowheads). (C) Electron micrograph of a cone pedicle in the OPL showing GluK5 localization (arrowheads) in processes of OFF-cone bipolar cells. (D—F) Electron microscopic analysis of GluK5 distribution in the IPL. In the outer part of the inner plexiform layer GluK5 labeling is found in processes postsynaptic to OFF-cone bipolar cells (D). In the inner part of the inner plexiform layer labeling is seen in processes postsynaptic to ON-cone bipolar cells (E) and to rod bipolar cells (F). The arrows point to the presynaptic ribbon, the presynaptic terminals are surrounded with red dotted lines; the postsynaptic GluK5 positive elements are visualized with blue dotted lines. For better visualization in the electron micrographs presynaptic elements are visualized with blue and postsynaptic elements with red dotted lines. H = horizontal cell, RS = rod spherule, CP = cone pedicle, Off-CBP = off-cone bipolar cell, On-CBP = on-cone bipolar cell, RBP = rod bipolar cell. All scale bars: 100 nm.

Ribbon synapses are formed between photoreceptors in the OPL and bipolar cells in the IPL [55–58]. They are named after the presence of a large electron dense band adjacent to the active zone of the synapse. To confirm the ribbon-associated localization of GluK5, we next performed pre-embedding EM immunostaining. We experienced that antigenicity to GluK5 decreased dramatically when using fixative solutions containing glutaraldehyde that, in turn, is required for good structural preservation in fine-structural studies. Fig 2 represents a compromise, showing specific immunostaining. Although fine-structural preservation is not optimal, the tissue components can still be identified. The two representative high-power electron micrographs shown (Fig 2A and 2B) illustrate the typical staining pattern for GluK5 that we observed in the OPL. The labeling surrounds the synaptic ribbon of rod photoreceptor terminals and appears to be restricted to the ribbon. Importantly, our results also revealed that presynaptic GluK5 labeling was restricted to rod photoreceptor terminals and spared cone photoreceptors, another class of mammalian photoreceptors (Fig 2A–2C). Cone photoreceptors are different from rod photoreceptors by exhibiting approximately 30 synaptic ribbons per terminal [59, 60]. In addition to ribbon synapses, cone photoreceptors exhibit a second type of synaptic contact, the so-called basal synapse. We observed on the ultrastructural level postsynaptic labeling for GluK5 at these synapses, where OFF-cone bipolar cells make flat contacts with the cone terminals (Fig 2C). This finding supports our light microscopy PNA GluK5 co-immunolabeling analysis revealing a close association of cone pedicles and GluK5. Together, these results further support our finding that the vast majority of anti-GluK5 labeling in the OPL is located to the presynaptic ribbon structure of rod photoreceptor terminals.

We also analyzed the localization of GluK5 in the IPL by EM and found most of the labeling localized to postsynaptic elements of both conventional synapses and ribbon synapses. Labeling was mainly found at processes presynaptic to OFF-cone bipolar cells in the outer part of the IPL, to processes in the inner part of the IPL postsynaptic to rod bipolar cells (Fig 2F) and rarely to presynaptic elements of ON-cone bipolar cells (Fig 2E). Interestingly, in cases where immunolabelling was located directly opposite to a bipolar cell ribbon, we found GluK5 immunoreactivity only in one of the two postsynaptic elements of the dyad, independent of the type of bipolar cell under study. Importantly, we failed to identify presynaptic labeling within the IPL. Neither conventional nor ribbon synapses exhibited anti-GluK5 labeling. From these data we conclude that GluK5 labeling in the IPL of the mouse retina is localized post-synaptically.

### Altered ribbon structure in the OPL in mutants deficient in GluK5

**Fig 3:**
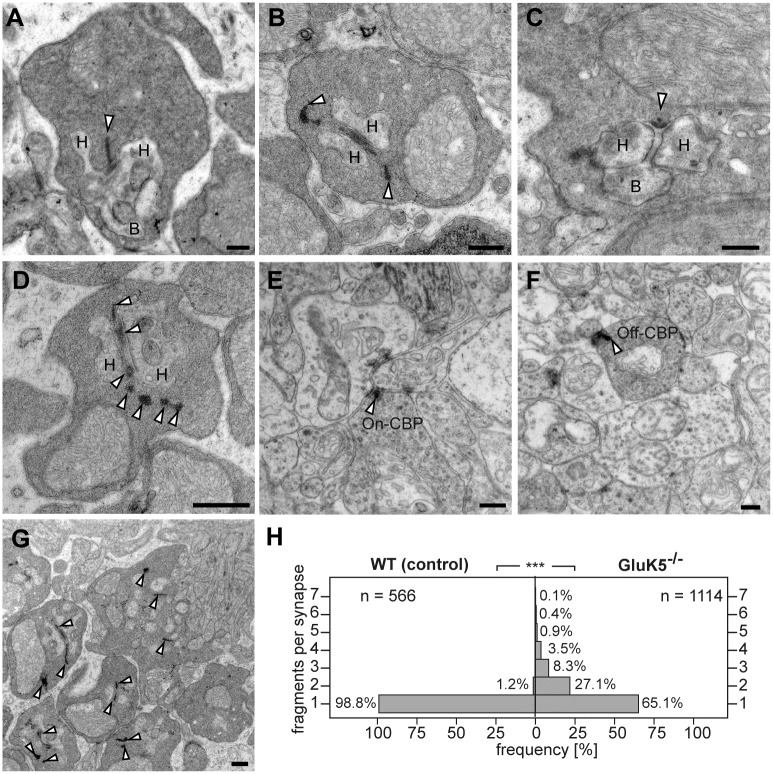
The absence of GluK5 disrupts the normal organization of presynaptic rod photoreceptor ribbons. The electron micrographs show sections through rod ribbon synapses in the outer plexiform layer of wild-type (A) and GluK5-knockout retinae (B-D, G). Wild-type terminals (A) show normal synaptic architecture with a synaptic ribbon anchored to the presynaptic membrane and with postsynaptic elements formed by processes of horizontal and bipolar cells. (B-D) Rod terminals in adult GluK5-/- mice showing “disintegrated” synaptic ribbons. In some cases ribbon material seems to be free-floating within the rod terminal (B and D, arrowheads). (E –F) The synaptic ribbon structure is not affected at synapses in the IPL as demonstrated for ribbons of ON-cone (E) and OFF-cone (F) bipolar cells. The lower magnification (G) shows that most of rod photoreceptor ribbons are affected in the GluK5 knockout retina. (H) Histogram showing the number of ribbon fragments at rod photoreceptor terminals in GluK5 knockout and wild type animals. n = number of presynaptic elements analysed. ***P < 0,001 Mann-Whitney-U-Test. H = horizontal cell, B = Bipolar cell, Off-CBP = off-cone bipolar cell, On-CBP = on-cone bipolar cell. All scale bars: 250 nm.

As we found GluK5 immunoreactivity in rod photoreceptor terminals localized to the synaptic ribbon we wondered whether GluK5 contributes to ribbon organization or function. Therefore, we performed an ultrastructural analysis of photoreceptor terminals in mice deficient of GluK5. When examining synapses in the OPL by standard EM we observed that virtually all synaptic terminals of rod photoreceptors exhibited massively altered synaptic ribbons (Fig 3B–3D). The electron-dense structure of the ribbon was disrupted or fragmented and very often appeared longer then in wild-type animals (Fig 3A). Furthermore, we observed in many synapses ribbon fragments being disintegrated within the synapse (Fig 3D). However, very often ribbon fragments appeared aligned and not randomly distributed (Fig 3C). When examining cone photoreceptor terminals of the OPL and ribbon synapses located within the IPL we did not observe these alterations of the synaptic ribbon structure in GluK5 deficient animals (Fig 3E and 3F), consistent with the absence of GluK5 immunostaining in these terminals. To substantiate our observations we quantified the number of ribbon fragments in rod presynaptic terminals of wild-type animals and GluK5-deficient mice (Fig 3G). We found in GluK5-deficient animals that 34.9% of the rod presynaptic terminals exhibited two or more ribbon fragments. In contrast, 98.8% of wild type rod terminals showed only a single synaptic ribbon structure. These data indicate that the loss of GluK5 might have an influence on the integrity of ribbon structure in rod ribbon synapses.

**Fig 4:**
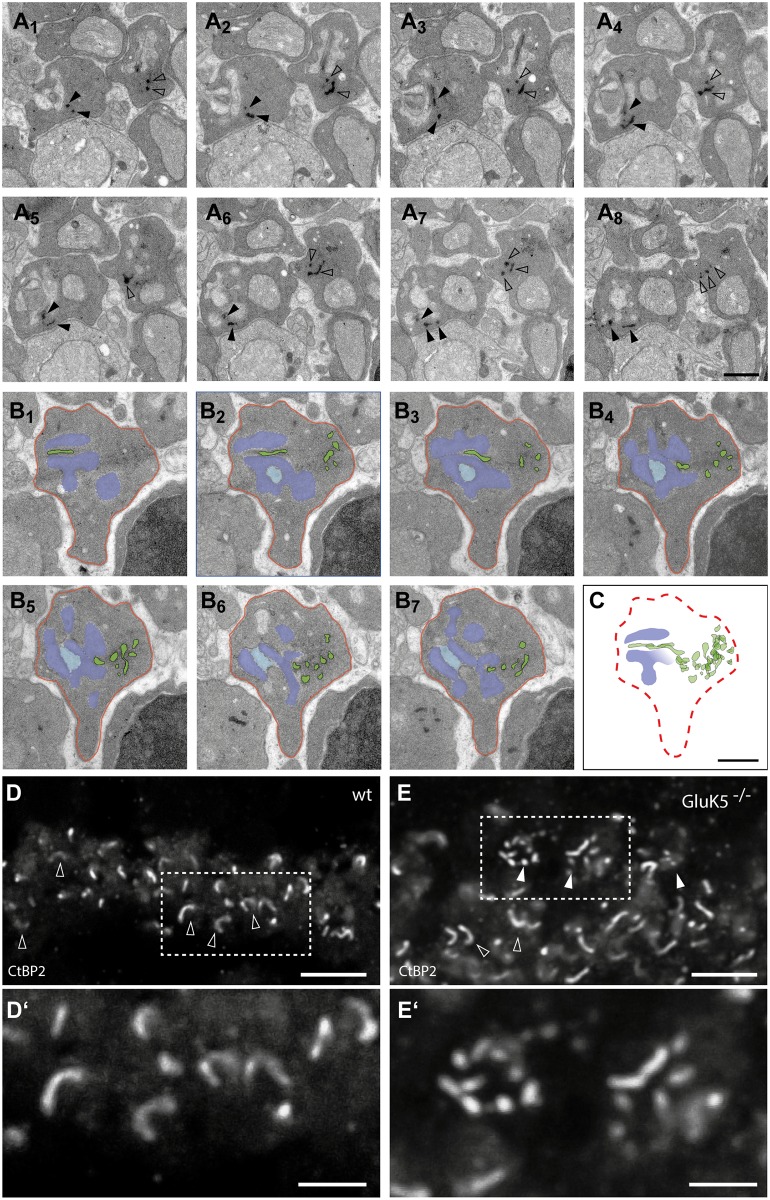
Electron microscopic analysis of synapses deficient in GluK5. (A_1_–A_8_) Electron micrographs showing a series of ultrathin sections taken through two rod photoreceptor synaptic terminals of a GluK5^-/-^ retina. The ultrastructural abnormalities in the course of the synaptic ribbons are clearly visible. In most sections the ribbons appear disintegrated (see arrowheads in A). (B_1_-B_7_) Electron micrographs showing a series of ultrathin sections taken through a rod photoreceptor synaptic terminal of a GluK5 knockout mouse. The ribbon and its fragments are visualized in green. For a better visualization the postsynaptic elements are colored in light and dark blue and the outline of the rod terminal is labelled in red. (C) Overlay of the colored green ribbon fragments. It is possible to follow the course of the ribbon through the matrix of the rod terminal. The overlay suggests that although the ribbon course is undulated its continuity might be conserved. (D and E) Immunohistochemistry for CtBP2 in wild-type and GluK5^-/-^ retinae. (D) Typical horseshoe shaped ribbons are observed (open arrowheads). (E) GluK5 deficient retinae reveal to a high degree punctuated immunolabeling (arrowheads). (D’ and E’) Higher magnification of tagged frames in D (D’) and E (E’). Scale bars (A—C): 500 nm; D and E: 5 μm.

Single sections do not provide information about the three dimensional course of the ribbon within a rod spherule. Therefore, we performed 3D reconstructions from serial thin sections (Fig 4A–4C). Two series of representative synapses are presented with one showing the original electron micrographs and one showing sections in pseudocolors for better identification of the ribbon structures (Fig 4B and 4C). In all analyzed synapses serial section analysis revealed that the rod photoreceptor ribbons in GluK5^-/-^ animals showed an extremely undulated course and aberrant orientation. Furthermore, it appeared that the ribbons were dramatically elongated and often fragmented (see also Fig 3G).

In order to verify the altered ribbon morphology seen on the ultrastructural level in GluK5^-/-^ retinae we next performed immunofluorescence staining for CtBP2, which also revealed an altered morphology of ribbons in GluK5^-/-^ animals (Fig 4D and 4E). Whereas in wild-type animals the majority of anti-CtBP2 labeling showed the typical horseshoe-like morphology of ribbons in the OPL, GluK5^-/-^ retinae rarely contained such structures (Fig 4E). We rather observed much more often short, fragmented structures in the OPL of GluK5-deficient mice when immunolabeling for CtBP2 (Fig 4E). These results support our hypothesis that GluK5 might play an important role in maintaining the structural integrity of the rod photoreceptor ribbon synapse.

## Discussion

In the present study we aimed to explore in more detail the localization and possible role of the high affinity ionotropic glutamate receptor GluK5 in the mouse retina.

Our analysis revealed unexpectedly evidence for a presynaptic localization of GluK5 in rod photoreceptors. Kainate receptors have already previously been described as being localized to presynaptic terminals outside of the retina [34–36] where they are activated by synaptically released L-glutamate [48, 61, 62] and are involved in both inhibition and facilitation of synaptic transmission [49, 63]. However, the only ionotropic glutamate receptor subunit that was found presynaptically in rod spherules was the C2 splice variant of GluN1, which additionally was also found being localized to cone pedicles [47]. The ultrastructural localization of GluN1 showed immunoreactivity at the collar of the invaginating synapse. In contrast, GluK5 appeared to be distributed in a different fashion as we observed GluK5 immunoreactivity being localized immediately adjacent to the ribbon structure of rod photoreceptors where glutamate is released. We could demonstrate the presynaptic localization by co-immunolocalization with the ribbon marker CtBP2 [49] and by immunolabeling at the ultrastructural level. Importantly, we could not detect any presynaptic immunoreactivity in cone pedicles. In contrast to these findings a previous study in the rat retina did not show presynaptic localization of GluK5 [64]. This discrepancy might result from the different antisera used in the two studies. It is for example well possible that the antisera exhibit different sensitivity. The antibody used in this study has already previously been used to demonstrate presynaptic localization of GluK5 in non-retinal neurons [34]. The strongest argument for the specificity of the antiserum used in this study comes from the lack of immunoreactivity in Western blotting experiments and immunohistochemistry when using tissue from animals deficient for GluK5 [42, 48]. Hence, it is also possible that the difference in immunoreactivity observed in the rat retina might reflect a difference in kainate receptor composition in rat and mouse. Beside photoreceptors, bipolar cells are the second class of cells exhibiting synaptic ribbons in the retina, which are localized within the IPL. Interestingly, we could not detect presynaptic localization of GluK5 in the terminals of these bipolar cells. Hence, it appears that kainate type glutamate receptors, or more generally ionotropic glutamate receptors, might play an important role in specialized neurons containing ribbon structures. Rod photoreceptors are responsible for vision at low light levels (scotopic vision). However, we could not see a change of GluK5 expression in rod photoreceptors when analyzing dark-adapted retinae (data not shown). Whether the localization of GluK5 to rod ribbon synapses reflects an individual role of GluK5 at these synapses or whether other kainate receptor family members are localized to ribbon synapses negative for GluK5 needs to be clarified. However, support for our hypothesis is coming from a recent study that demonstrated the two kainate receptor subunits GluK5 and GluK2 being localized to synaptic ribbons in functional outer hair cell (OHC) afferent synapses [65] pointing indeed to an important function of kainate type receptors at the ribbon structure.

For hippocampal neurons it has been speculated that presynaptic kainate receptors might maintain high levels of transmitter release during high-frequency transmission as it is necessary for the induction of long-term potentiation [49, 66]. This way they could participate in providing a presynaptic depolarization that facilitates the activation of voltage-gated Ca^2+^ channels. Furthermore, presynaptic kainate receptors might also gate directly Ca^2+^ fluxes [67, 68]. For mossy fiber synapses in the hippocampus it has been shown that Ca^2+^-induced Ca^2+^ release from intracellular stores is involved in the induction of mossy fiber LTP and that the trigger for this Ca^2+^ release is Ca^2+^ permeation through kainate receptors [69]. From these data one can postulate that presynaptic kainate receptors can initiate a signaling cascade involving the release of Ca^2+^ from intracellular stores that is important in both short-term and long-term plasticity [69]. Our findings of a presynaptic localization of GluK5 at rod photoreceptor synapses are in line with these results at non-retinal synapses. Furthermore, the regulation of Ca^2+^ release from internal stores via kainate receptors might provide an explanation for the present finding of GluK5 being associated with the synaptic ribbon.

Presynaptic kainate receptors may modulate Ca^2+^-release either in an ionotropic or in a metabotropic fashion. Interestingly, from the three subtypes of voltage gated L-type Ca^2+^ channels known to trigger glutamate release, the Cav1.4 subtype has been localized primarily to rod and bipolar cell terminals [70, 71]. Recently, it has been shown that the sites of calcium influx are co-localized with the synaptic ribbon [52] and that Ca^2+^ channels cluster near the ribbons [53, 54]. Furthermore, it has been shown that the alpha1 subunit of the Ca^2+^ channel colocalizes with RIBEYE [72, 73], the main structural protein of synaptic ribbons [49]. The close expression patterns of GluK5 and the alpha1 subunit of the Ca^2+^ channel (S1C and S1C’ Fig) support the hypothesis of a functional interaction. Outside of the retina a presynaptic synergistic interaction of kainate and metabotropic glutamate receptors with ryanodine receptors has also been postulated and shown to regulate Ca^2+^ signaling in mossy fiber boutons of adult rats [74]. In the retina the group III metabotropic glutamate receptor mGluR8 has been described presynaptically in rod photoreceptor terminals and assumed to modulate mainly L-type calcium channels [72]. Together with the findings that ryanodine receptors in mouse rod photoreceptors mediate the release of Ca^2+^ from the endoplasmic reticulum in proximity to the synaptic ribbon [75] this pathway could also be a possible function of GluK5 at ribbons. The precise mechanism by which retinal presynaptic GluK5 could trigger Ca^2+^ channels has remained elusive and needs to be studied.

To become functional high affinity kainate receptors have to form heterodimers with low affinity subunits (GluK1-GluK3) irrespective of whether they function as heteroreceptors or as autoreceptors. With our assays we were unable to detect any coexpression of GluK5 with an antibody recognizing the low affinity subunits GluK1-3. This discrepancy was not surprising and might be due to the different sensitivities of the antisera used, as to date, reliable antibodies recognizing all low affinity subunits are still not available yet. However, using multiplex RT-PCR on micro-dissected single rod photoreceptors co-expression of GluK5 with either GluK1 or Gluk2 could be demonstrated [76].

To verify the specificity of the antibody used in this study and to study the possible function of presynaptic GluK5 localized to rod ribbon synapses we also analyzed the ultrastructure of photoreceptor ribbon synapses in GluK5 deficient mice [42]. Intriguingly, we observed in *GluK5*^*-/-*^ animals an alteration of the integrity and size of rod photoreceptor ribbons. We found rod photoreceptor ribbons to a very high degree disrupted and appearing substantially larger compared to wild-type animals. Unfortunately, the partial fragmentation of the ribbons precluded a detailed quantitative analysis of ribbon length. Nevertheless, it is surprising that the elimination of a single glutamate receptor subunit affects the size and/or structure of the synaptic ribbon to such an extent. The strong alteration in synaptic ribbon shape and integrity in *GluK5*^*-/-*^ animals might reflect an additional unknown function of high affinity kainate receptors that might be different from its classical function. One might also speculate that the functional interaction of kainate receptors and calcium channels could determine and affect the size and shape of the synaptic ribbon. Evidence for this hypothesis comes from recent studies reporting that manipulating internal calcium levels resulted in structural changes of photoreceptor ribbons [73, 77, 78]. Furthermore, rod photoreceptors lacking the calcium channel Cavβ2 exhibit a complete disappearance of synaptic ribbons although synaptic vesicles and their density appeared normal [13]. In line with these findings, it has been demonstrated in hair-cell synapses that the size of mature synaptic ribbons positively correlates with calcium influx [79, 80]. Importantly, this effect appears to be reversed at early developmental stages, as the acute block of Ca^2+^ influx through the L-type calcium channels Cav1.3a during a critical period of hair-cell development leads to an increase in ribbon size [81]. It has been speculated that the Ca^2+^ influx through L-type Calcium channels may regulate the assembly of the Ribeye protein, thereby affecting the size and morphology of synaptic ribbons in hair cells. The *GluK5*^*-/-*^ animals used in this study are constitutive knockout mice. Therefore, GluK5 is already absent during early development and synaptogenesis and could result in an alteration of ribbon structures similar to that in hair cells due to the absence of a functional interaction with calcium channels. Based on these findings one may speculate that a cooperation of kainate receptors and calcium channels may affect ribbon size via Ca^2+^ influx. However, a potential interaction of GluK5 with Ca^2+^ channels in rod photoreceptor ribbon synapses and the precise role of Ca^2+^ on the structure and integrity of the ribbon in these synapses needs to be clarified in detail in future studies.

Besides its presynaptic localization we found GluK5 also localized to postsynaptic structures. We found GluK5 immunoreactivity postsynaptically in processes of OFF-bipolar cells and within the IPL in processes postsynaptic to ON and OFF cone bipolar cells as well as rod bipolar cells. These latter processes in the IPL are presumably belonging to ganglion cells and amacrine cells. This observation is in agreement with recent findings demonstrating GluK4 and GluK5 as being expressed in ganglion cells in the developing and adult mouse retina and being localized to these processes [82].

In conclusion we provide new data for a presynaptic localization of the iontropic glutamate receptor GluK5 in the mouse retina localized to rod photoreceptor ribbon synapses and additional evidence for an important role of GluK5 in preserving the structural integrity of the synaptic ribbon. The presynaptic localization of GluK5 in rod photoreceptors strengthens the increasing evidence of a fundamental regulatory function of kainate receptors in presynaptic compartments.

## Supporting information

S1 Fig Immunohistochemical analysis of the mouse retina using an anti-GluK5 antibody. (A) Immunofluorescence analysis showing strong, punctate anti-GluK5 staining in the outer and inner plexiform layer of the wildtype mouse retina. (A’) No GluK5 labeling is present in the GluK5-/- retina. (B and B’) PNA-labeling (green) for the identification of cone terminals and their contacts with OFF cone bipolar cell dendrites and immunolabeling for GluK5 show a close association of GluK5 with PNA labeled cone pedicles (arrowheads) in WT animals. The asterisk marks a blood vessel. (C-C’) Double-labeling for anti-GluK5 (red) and anti-CACNA1F (green) showing adjacent localization in WT retinae. Scale bars: A—A’: 10 μm; B—B’: 5 μm; C –C’: 2 μm.
(TIF)
