# Supplementary figures and images for: Presynaptic localization of GluK5 in rod photoreceptors suggests a novel function of high affinity glutamate receptors in the mammalian retina
Source: PLoS One. 2017 Feb 24;12(2):e0172967. doi: 10.1371/journal.pone.0172967 (PMC5325551; doi:10.1371/journal.pone.0172967)

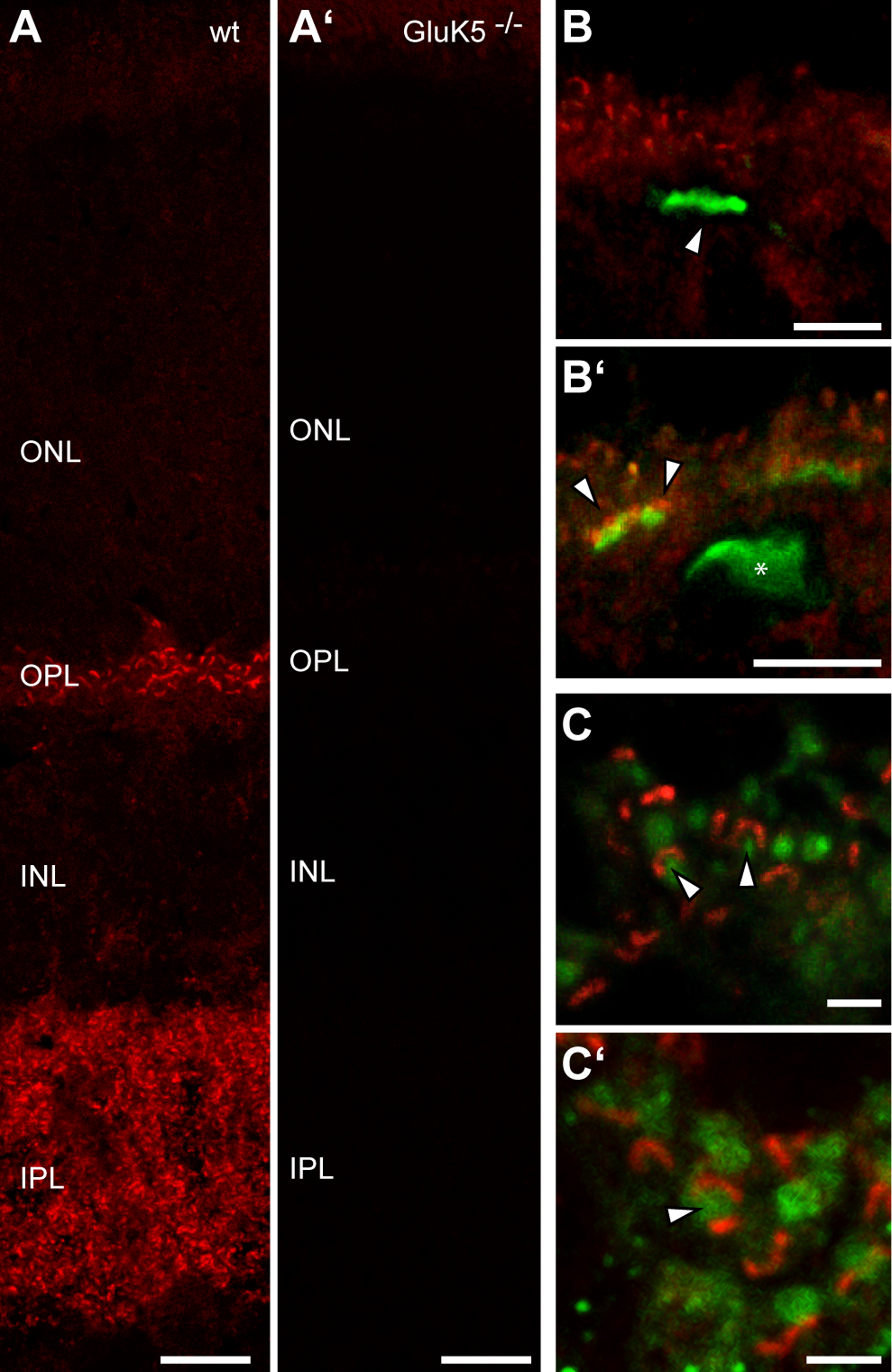

Supplement: S1 Fig — (A) Immunofluorescence analysis showing strong, punctate anti-GluK5 staining in the outer and inner plexiform layer of the wildtype mouse retina. (A’) No GluK5 labeling is present in the GluK5-/- retina. (B and B’) PNA-labeling (green) for the identification of cone terminals and their contacts with OFF cone bipolar cell dendrites and immunolabeling for GluK5 show a close association of GluK5 with PNA labeled cone pedicles (arrowheads) in WT animals. The asterisk marks a blood vessel. (C-C’) Double-labeling for anti-GluK5 (red) and anti-CACNA1F (green) showing adjacent localization in WT retinae. Scale bars: A—A’: 10 μm; B—B’: 5 μm; C –C’: 2 μm. (TIF) [file pone.0172967.s001.tif]
